# Nelson Bay Orthoreovirus cell attachment protein σC determines strain-specific differences in infectivity and pathogenesis

**DOI:** 10.1371/journal.ppat.1014409

**Published:** 2026-08-03

**Authors:** Takahiro Kawagishi, Yusuke Sakai, Hiroya Oki, Ryotaro Nouda, Yuta Kanai, Kazuki Kawahara, Shota Nakamura, Masayuki Shimojima, Masayuki Saijo, Yoshiharu Matsuura, Takeshi Kobayashi

**Affiliations:** 1 Department of Virology, Research Institute for Microbial Diseases (RIMD), The University of Osaka, Osaka, Japan; 2 Department of Infectious Disease Pathology, National Institute of Infectious Diseases, Japan Institute for Health Security, Tokyo, Japan; 3 Graduate School of Pharmaceutical Sciences, The University of Osaka, Osaka, Japan; 4 Department of Infectious Metagenomics, Research Institute for Microbial Diseases (RIMD), The University of Osaka, Osaka, Japan; 5 Center for Advanced Modalities and DDS (CAMaD), The University of Osaka, Osaka, Japan; 6 Graduate School of Drug Discovery Sciences, Osaka Metropolitan University, Osaka, Japan; 7 Department of Virology I, National Institute of Infectious Diseases, Japan Institute for Health Security, Tokyo, Japan; 8 Public Health Office, Health and Welfare Bureau, City of Sapporo, Hokkaido, Japan; 9 Laboratory of Virus Control, Research Institute for Microbial Diseases (RIMD), The University of Osaka, Osaka, Japan; 10 Center for Infectious Disease Education and Research (CiDER), The University of Osaka, Osaka, Japan; National University of Singapore, SINGAPORE

## Abstract

Nelson Bay orthoreovirus (NBV) was initially discovered in a bat sample but has since been isolated from patients with acute respiratory tract diseases. Accumulating reports of NBV isolation from patients with respiratory tract viral infections suggest that NBV is able to transmit and cause disease in humans. However, the underlying molecular mechanisms remain unclear. We previously established a reverse genetics system for NBV Miyazaki-Bali/2007 (MB) strain isolated from a patient with an acute respiratory tract disease. We found that the fusion-associated small transmembrane protein (FAST)—which is necessary for syncytium formation—and cell attachment protein σC play crucial roles in MB virulence; however, whether these gene products determine the strain-specific difference in NBV virulence remains unclear. Therefore, here, we compared the virulence of the MB strain with that of the NBV strain isolated from a bat sample (NelB strain). We found that the NelB strain did not cause a virulent phenotype in the mouse model. Using reverse genetics, we found that the S1 gene segment correlates with the virulent phenotypes of NBV strains. Moreover, among the three proteins encoded by the S1 gene segment, structural protein σC, but not nonstructural proteins FAST or p17, contributed to the difference in virulence *in vivo*. Further analysis using a panel of σC mutant viruses showed that the middle body domain in σC was involved in the different virulent phenotypes, rather than the C-terminal head domain, which contains a putative receptor-binding domain. These results provide new insights into the mechanisms underlying NBV transmission and pathogenesis.

## Author summary

Bats are rich sources of DNA and RNA viruses including RNA viruses that are highly pathogenic to humans. The virulent phenotype of bat-borne viruses in humans draws attention to the molecular mechanisms underlying the transmission and pathogenesis of bat-borne viruses. Nelson Bay orthoreovirus (NBV) is a bat-borne orthoreovirus that has been isolated from patients with acute respiratory tract diseases; however, the relationship between the differences in viral proteins and NBV transmission and pathogenesis in humans remain unclear. In this study, we performed a comparative analysis of NBVs isolated from a bat and human patient. We found that the difference in the amino acid of the body domain of the cell attachment protein σC contributes to the different NBV virulent phenotypes. This finding indicates the critical role of the cell attachment protein σC in cross-species transmission of NBV.

## Introduction

An increasing number of reports demonstrate that bats are a rich source of DNA and RNA viruses from different families (database of bat-associated viruses) [1]. Among these, some bat-borne RNA viruses (e.g., Marburg virus, Ebola virus, Hendra virus, Nipah virus, Severe acute respiratory syndrome coronavirus) infect and cause lethal diseases in humans [2], although the bats themselves show minimal signs of disease from these viral infections. Recent findings indicate that the host-specific differences in pathogenicity are primarily driven by the distinct immune responses between bats and other mammals [3–5]. Comparative analysis between viruses isolated from bats and other mammalian species would provide helpful insight into understanding the molecular mechanisms underlying virulence and viral replication of bat-borne viruses.

Nelson Bay orthoreovirus (NBV) is a member of the genus Orthoreovirus in the family *Spinareoviridae*. NBV has been isolated from bat, monkey, and human specimens and bat flies. The first NBV strain Nelson Bay (hereafter referred to as NelB) was isolated in 1968 from the blood sample of a fruit bat [6,7]. However, NBV was not linked to diseases in humans until the isolation of the Melaka (Mel) strain from a patient suffering from acute respiratory disease in Malaysia in 2007 [8]. Subsequently, several NBV strains have been isolated from humans with respiratory tract infections, bats, monkey samples, and bat flies in Asian and African countries (e.g., Malaysia, Indonesia, China, Philippines, Japan, Uganda, and Zambia) [9–24]. We isolated and characterized the NBV Miyazaki-Bali/2007 (MB) strain in Japan from a patient who returned from Indonesia and developed an acute respiratory tract disease [15]. Whether the recently isolated NBV strains exhibit different phenotypes compared with those of the original NelB strain, and whether these NBV strains from bats are transmitted to and persist in human populations need to be studied in depth.

The virulence of NBV strains has been evaluated in mouse models after intranasal inoculation [22,23,25,26]. We previously showed that intranasal infection with the MB strain led to a lethal outcome in a mouse model [26]. Similarly, NBV strains recently isolated from bat samples (e.g., Samal-24 and Paguyaman ORV13–27) caused lethal infections in a mouse model [23,25]. Thus, established mouse models are available for comparative analysis of NBV virulence.

NBV is a non-enveloped virus with a 10-segmented double-stranded RNA genome. Among the 10 gene segments, the S1 gene segment is the most genetically diverse among NBV strains [8,10,13,15,19,21,22,24]. The S1 gene segment contains three partially overlapping open reading frames (ORFs), which encode two nonstructural proteins (fusion-associated small transmembrane protein [FAST] and p17) and one structural protein (σC) in an out-of-frame manner [27,28]. These three proteins play vital roles in NBV life cycle. FAST expression induces membrane fusion with neighboring cells and syncytia formation and enhances viral replication in cell lines [29]. The other nonstructural protein p17 localizes in the nucleus of infected cells and regulates the cell–cell fusion mediated by FAST in a specific bat cell line [30]. The structural protein σC is a spike protein that constitutes the surface of the virion. It forms a homotrimer with three domains, namely, the N-terminal foot, central body, and C-terminal head and is involved in cell attachment and entry [31].

We previously used plasmid-based reverse genetics for MB strain and examined the roles of FAST, p17, and σC from a human isolate in viral replication and pathogenesis *in vivo* [29–31]. Notably, we rescued recombinant virus strains that were deficient in expressing FAST, p17, or σC (rsMB-ΔFAST, rsMB-Δp17, and rsMB-ΔσC) [29–31]. Comparative analyses of these knockout viruses with the wild-type rsΜΒ showed that rsMB-ΔFAST and rsMB-ΔσC lost their virulence in the mouse model for acute respiratory tract infection, whereas rsMB-Δp17 did not [29–31]. These data show that FAST and σC from the MB strain contribute to pathogenesis in the mouse model; however, whether these gene products (*i.e*., FAST or σC) determine the strain-specific difference in NBV virulence remains unclear.

In this study, we compared the pathogenesis of the NBV strains isolated from a bat (NelB strain) in 1968 and human patient (MB strain) in 2007. We generated a panel of chimeric viruses with S1 gene products between MB and NelB strains. We evaluated the roles of FAST, p17, and σC from the NelB strain in viral replication and virulence. Our findings revealed novel mechanisms underlying NBV infection in humans.

## Results

### Mouse model-based comparative analysis of NBV strains isolated from bat and human samples

**Fig 1:**
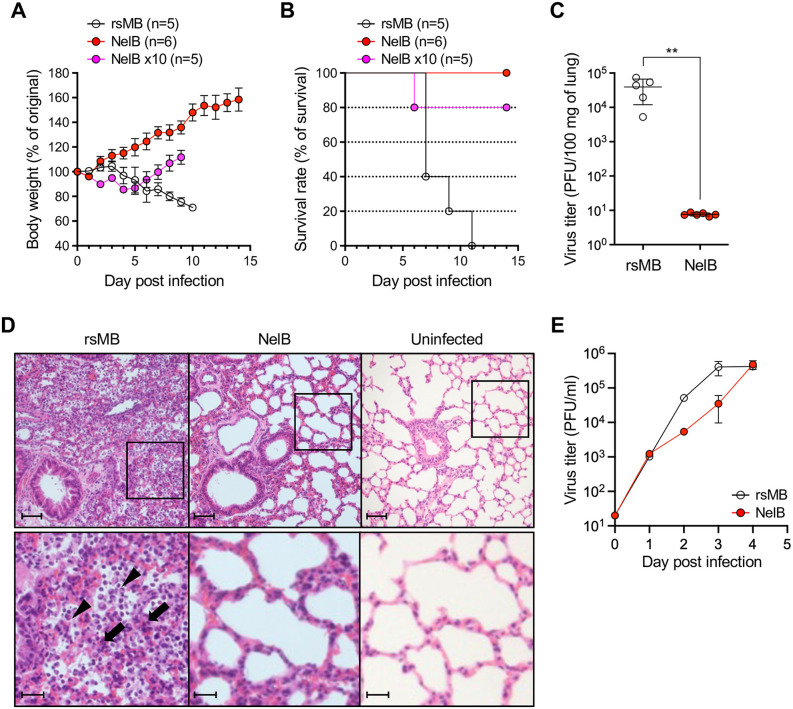
Comparative analysis of NBV strains isolated from bat and human samples using a mouse model. (A) Body weight loss and (B) survival curve of mice infected with NBV rsMB or NelB strains. Four-week-old C3H mice were intranasally inoculated with 2 × 10^5^ PFU/head of rsMB (n = 5) or NelB (n = 6), or 2 × 10^6^ PFU/head of NelB (n = 5) and monitored for two weeks. (A) Body weight is expressed as the relative value of current weight to original body weight at the time of inoculation. Data represent mean scores with standard deviations. (B) Percentage of the living mice are shown for survival curve. (C) Virus titer in the lungs of infected mice. Four-week-old C3H mice were intranasally inoculated with 2 × 10^5^ PFU/head of rsMB (n = 5) or NelB (n = 6) and euthanized at 6 days post-infection. Virus titer in the lung homogenates was determined using plaque assay. Data represent mean scores with standard deviations. Statistical significance is indicated as **P < 0.01. (D) Histopathological analysis of lungs from mice infected with rsMB or NelB and from uninfected controls. The top row shows representative lung histopathology (Scale bars = 50 μm). The bottom row shows higher-magnification images corresponding to the boxed regions in the top row. Inflammatory cell infiltration into the alveolar space and alveolar wall is indicated by arrowheads and arrows, respectively (scale bars = 20 μm). Four-week-old C3H mice were intranasally inoculated with 2 × 10^5^ PFU/head of rsMB or NelB and euthanized at 6 days post-inoculation. Lungs were fixed in 10% formalin and stained with hematoxylin and eosin. (E) Growth kinetics of rsMB and NelB in L929 cells. L929 cells were infected with the viruses at MOI of 0.01 PFU/cell and harvested at indicated time points. Virus titer was determined by plaque assay. Data represents mean scores with standard deviations.

We previously developed a mouse model of acute respiratory tract infection for NBV using the pathogenic strain MB [26]. The intranasal inoculation of MB caused a lethal respiratory tract infection in C3H mice. This infection was characterized by a higher virus titer in the lungs compared with that in other tissues. In contrast, the pathogenicity of the NelB strain remains unknown. Hence, we characterized its virulence by intranasally inoculating C3H mice with 2 × 10^5^ PFU/head of NelB strain and compared the virulence with that of the MB strain. The mice infected with the wild-type recombinant MB strain (rsMB), which was generated through reverse genetics, showed severe body weight loss, and all mice died by 11 days post-inoculation (Fig 1A and 1B). This result was consistent with the previously reported findings of the study on MB strain [26]. In contrast, all mice infected with the NelB strain survived without any body weight loss. Moreover, four out of five mice (80%) survived after being inoculated with a 10-fold higher dose (2 × 10^6^ PFU/head) of NelB, although body weight loss was observed (Fig 1A and 1B). We compared the viral load in the lungs of the inoculated mice by measuring the viral titer in the lungs of the rsMB- and NelB-infected mice at 6 days post-inoculation. As previously reported, the lungs of the rsMB-infected mice showed a titer of approximately 10^4^ PFU/100 mg of the lung; however, the lungs of the NelB-infected mice showed a lower titer than the limit of detection in this assay (Fig 1C). Additionally, we performed histopathological analysis to compare the lung damage in the rsMB- or NelB-infected mice with that of the control uninfected mice. Hematoxylin and eosin (HE) staining of the lungs from the infected mice showed that rsMB caused severe pneumonia characterized by massive inflammatory cell infiltration into the alveolar space and alveolar wall (Fig 1D). In contrast, NelB infection caused only slight inflammatory cell infiltration into the alveolar walls (Fig 1D). Furthermore, similar to the uninfected mouse lungs, NelB did not cause monocyte accumulation (Fig 1D). Growth kinetics of the viruses in murine L929 cells showed that NelB replicated at a similar titer to that of rsMB, which suggests that the NelB stock used in the animal experiment is capable of replicating in a cultured cell line (Fig 1E). Collectively, these data indicate that the NBV strains isolated from human (rsMB) and bat (NelB) samples differ in their capacity to cause lethal infection in the mouse model.

### NBV S1 gene segment is a virulence determinant *in vivo*

**Fig 2:**
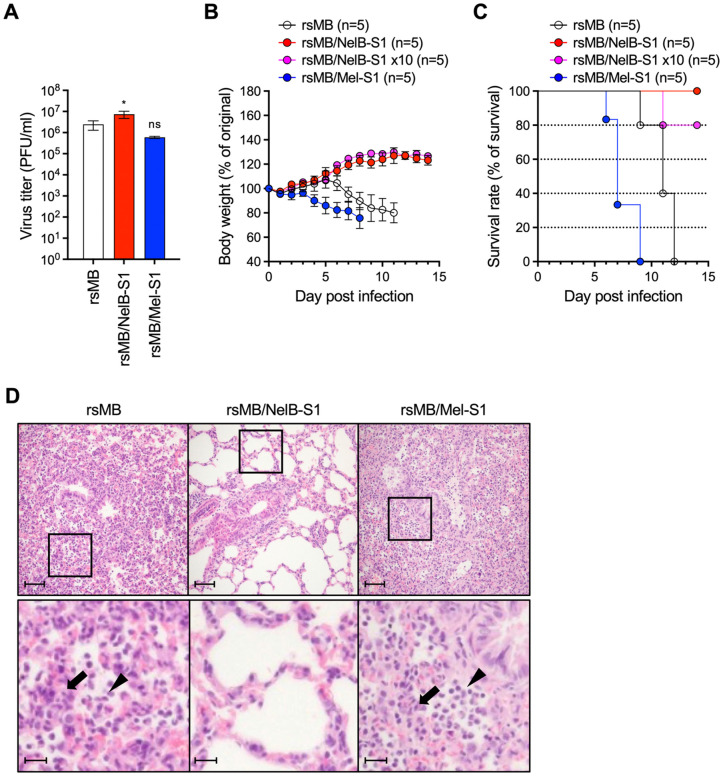
S1 gene segment of NBV is a virulence determinant *in vivo.* (A) Replication of S1 monoreassortant viruses in L929 cells. L929 cells were infected with the viruses at MOI of 0.01 PFU/cell and harvested at 3 days post infection. Virus titer in the cell lysates was determined using plaque assay. Data represents mean scores with standard deviation. Statistical significance is indicated as *P < 0.05 and ns: not significant. (B) Body weight loss and (C) survival curve of mice infected with the S1 monoreassortant viruses. Four-week-old C3H mice were intranasally inoculated with 2 × 10^5^ PFU/head of rsMB (n = 5), rsMB/NelB-S1 (n = 5), or rsMB/Mel-S1 (n = 5) or 2 × 10^6^ PFU/head of rsMB/NelB-S1 (n = 5) and monitored for two weeks. (B) Body weight is expressed as the relative value of current weight to original body weight at time of inoculation. Data represent mean scores with standard deviations. (C) Percentage of living mice are shown for survival curve. (D) Histopathological analysis of lungs from mice infected with rsMB, rsMB/NelB-S1, or rsMB/Mel-S1. The top row shows representative lung histopathology (Scale bars = 50 μm). The bottom row shows higher-magnification images corresponding to the boxed regions in the top row. Inflammatory cell infiltration into the alveolar space and alveolar wall is indicated by arrowheads and arrows, respectively. (scale bars = 20 μm). Four-week-old C3H mice were intranasally inoculated with 2 × 10^5^ PFU/head of rsMB, rsMB/NelB-S1, or rsMB/Mel-S1 and euthanized at 6 days post inoculation. Lungs were fixed with 10% formalin and stained with hematoxylin and eosin.

To identify the gene segments associated with the difference in virulence in the NBV mouse model, we compared the nucleotide and amino acid sequence identities between rsMB and NelB strains. Among the 10 gene segments, the S1 gene segment showed the lowest nucleotide identity (57.9%) compared with those of the other nine gene segments (79.8–87.5%) (Table 1). The three proteins encoded in the S1 gene segment (FAST, p17, and σC) were less homologous (72.6, 52.9, and 43.6%, respectively) than the other nine proteins (91.5–98.1%) (Table 1). This suggests that the S1 gene segment is involved in the different virulence phenotypes between the MB and NelB strains. To evaluate the contribution of the S1 gene segment on strain difference in pathogenesis, we used S1 monoreassortant viruses generated previously [31]. rsMB/NelB-S1 harbors the S1 gene segment from the NelB strain, whereas rsMB/Mel-S1 contains the S1 gene segment from the Mel strain, (the first strain isolated from a patient with acute respiratory tract illness). These S1 monoreassortant viruses replicated efficiently in the L929 cells (Fig 2A). We inoculated C3H mice with 2 × 10^5^ PFU/head of the viruses via the intranasal route and compared the parameters such as body weight loss, survival rate, and viral titer in the lungs. Like rsMB-infected mice, the rsMB/Mel-S1-infected mice developed severe body weight loss and died by 9 days post-infection (Fig 2B and 2C). However, the mice infected with rsMB/NelB-S1 did not show body weight loss and survived (Fig 2B and 2C). As observed for the NelB strain, a 10-fold higher dose of rsMB/NelB-S1 (2 × 10^6^ PFU/head) killed only one out of five mice (20%) and did not cause body weight loss in most mice (Fig 2B and 2C). Histopathology showed severe inflammatory reactions in the lungs of the rsMB/Mel-S1-infected mice similar to that observed after rsMB infection; however, the rsMB/NelB-S1-infected mice did not show such inflammatory reactions (Fig 2D). These data indicate that the introduction of the S1 gene segment of the NelB strain into the MB strain background reduced the virulence of the MB strain. Additionally, it suggests that the S1 gene segment is a critical determinant in the virulent phenotype of NBV strains.

**Table 1:** Nucleotide and amino acid identities of NBV MB and NelB strains.

Gene Segment (Protein)	Nucleotide identity (%)	Amino acid identity (%)
L1 (λC)	81.9	92.3
L2 (λB)	83.4	96.3
L3 (λA)	84.3	96.7
M1 (μA)	83.1	92.1
M2 (μB)	79.8	96.6
M3 (μNS)	82.6	91.5
S1 (FAST)	57.9	72.6
S1 (p17)		52.9
S1 (σC)		43.6
S2 (σA)	86.7	98.1
S3 (σNS)	87.5	96.7
S4 (σB)	83.6	93.4

### Cell attachment protein σC determines difference in virulence phenotypes between NelB and MB strains

**Fig 3:**
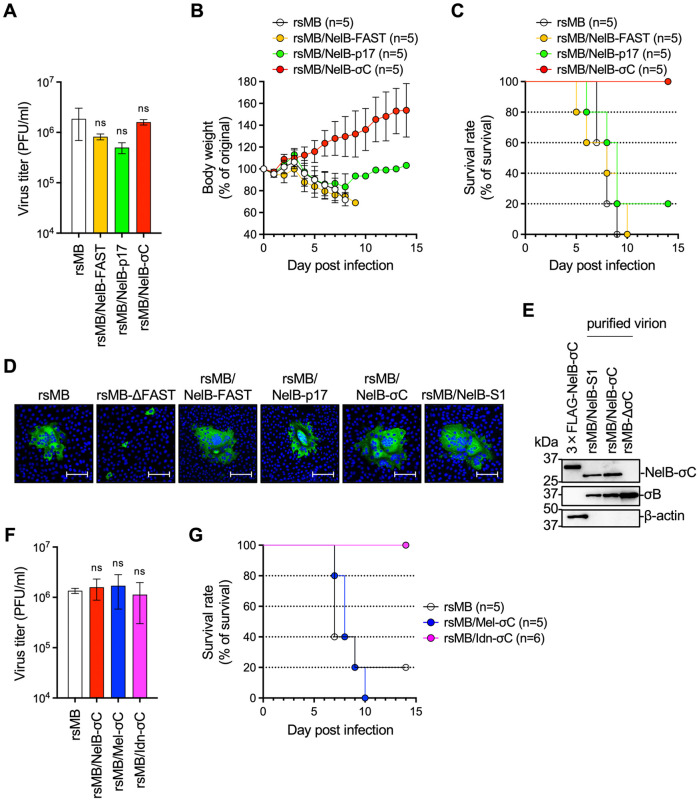
Cell attachment protein σC determines the difference in virulence phenotypes between NelB and rsMB strains. (A) Replication of chimeric viruses of S1 gene products in L929 cells. L929 cells were infected with the viruses at MOI of 0.01 PFU/cell and harvested at 3 days post infection. Viral titer in cell lysates were determined using plaque assay. Data represent mean scores with standard deviation. Statistical significance is indicated as ns: not significant. (B) Body weight loss and (C) survival curve of mice infected with chimeric viruses of S1 gene products. Four-week-old C3H mice were intranasally inoculated with 2 × 10^5^ PFU/head of rsMB (n = 5), rsMB/NelB-FAST (n = 5), rsMB/NelB-p17 (n = 5), or rsMB/NelB-σC (n = 5) and monitored for two weeks. (B) Body weight is expressed as relative value of current weight to original body weight at time of inoculation. Data represent mean scores with standard deviations. (C) Percentage of living mice are shown for survival curve. (D) Cell fusion induced by rescued viruses. Vero cells were infected with the viruses at MOI of 0.1 PFU/cell and fixed with formalin at 12 h post-inoculation. Virus-infected cells and nuclei were stained with antisera against MB strain and DAPI, respectively. Scale bar = 100 μm. (E) Detection of structural proteins in purified virions. Purified virions (rsMB/NelB-S1, rsMB/NelB-σC, or rsMB-ΔσC) were subjected to SDS-PAGE. Viral structural proteins σC and σB or cellular β-actin were detected using specific antibodies. (F) Replication of σC chimeric viruses in L929 cells. L929 cells were infected with the viruses at MOI of 0.01 PFU/cell and harvested at 3 days post infection. Viral titer in cell lysates were determined using plaque assay. Data represent mean scores with standard deviation. Statistical significance is indicated as ns: not significant. (G) Survival curve of mice infected with NBV rsMB, rsMB/Mel-σC, or rsMB/Idn-σC strains. Four-week-old C3H mice were intranasally inoculated with 2 × 10^5^ PFU/head of rsMB (n = 5), rsMB/Mel-σC (n = 5), or rsMB/Idn-σC (n = 6) and monitored for two weeks. Percentage of the living mice are shown for survival curve.

Based on the observations in the mouse model, we previously reported that among the three viral proteins (FAST, p17, and σC) encoded in the NBV S1 gene segment, the nonstructural protein FAST and structural protein σC of the MB strain independently contribute to pathogenesis although p17 is not involved in virulence [29–31]. In the present study, to explain the difference in virulence between the MB and NelB strains, we clarified which of these viral protein(s) are minimally required by generating chimeric viruses that possess FAST, p17, or σC of the NelB strain in the genetic background of the rsMB strain (rsMB/NelB-FAST, rsMB/NelB-p17, and rsMB/NelB-σC, respectively) (S1 Fig). We found that these viruses replicated to a similar extent as that of the rsMB strain (Fig 3A). We intranasally inoculated C3H mice with these chimeric viruses to compare the virulence in the mouse model. Of note, rsMB/NelB-FAST and rsMB/NelB-p17 caused body weight loss and killed 100% (five out of five) and 80% (four out of five) of the infected mice, respectively (Fig 3B and 3C). However, the mice infected with rsMB/NelB-σC survived without developing lethal respiratory infection (Fig 3B and 3C). This suggests that among the three viral proteins encoded in the S1 gene segment, σC is associated with the difference in virulence between NBV isolated from humans and bats in the mouse model.

FAST and σC proteins are required for the lethal infection of the MB strain *in vivo*. Therefore, we examined the production of FAST and σC by rsMB/NelB-S1 and rsMB/NelB-σC to exclude the possibility that these viruses do not express functional FAST and σC, which led to avirulent phenotypes *in vivo*. To examine cell–cell fusion activity, we inoculated Vero cells with rsMB/NelB-S1, rsMB/NelB-σC, or control viruses (rsMB and rsMB-ΔFAST) along with other S1 chimeric viruses (rsMB/NelB-FAST and rsMB/NelB-p17). As previously reported, rsMB-ΔFAST did not form syncytia in infected cells because of a lack of FAST expression (Fig 3D). In contrast, we observed syncytium formation by rsMB/NelB-S1 and rsMB/NelB-σC, as was produced by rsMB strain. Additionally, we observed syncytia in the cells infected with rsMB/NelB-FAST or rsMB/NelB-p17 (Fig 3D). This indicates that rsMB/NelB-S1 and rsMB/NelB-σC express a functional FAST protein in infected cells. Next, we prepared purified virions using a CsCl gradient and detected NelB σC in the purified virions. Western blotting showed that rsMB/NelB-S1 and rsMB/NelB-σC contained σC proteins in the purified virions (Fig 3E). Thus, the avirulent phenotypes of rsMB/NelB-S1 and rsMB/NelB-σC cannot be attributed to a defect in FAST or σC protein expression.

To extend our understanding of the relationship between σC and its virulence *in vivo*, we constructed two additional rescue plasmids for chimeric viruses with σC from NBV strains isolated from human (Melaka strain) or bat (Indonesia/2010 strain) samples. As the nucleotide sequences of σC are divergent among the NBV strains, we selected the Indonesia/2010 strain, which was isolated from a bat and showed the highest nucleotide identity for the S1 gene segment to that of the NelB strain (77%) [17]. Using these plasmids, we generated rsMB/Mel-σC and rsMB/Idn-σC (Fig 3F). Although the rsMB/Mel-σC-inoculated C3H mice died after intranasal inoculation, the rsMB/Idn-σC-inoculated mice survived (Fig 3G). These results indicate that the difference in σC protein between NBV isolated from bat and human samples is associated with an NBV virulent phenotype *in vivo*.

### Functional difference between σC from MB and NelB strains in infection and cell attachment

**Fig 4:**
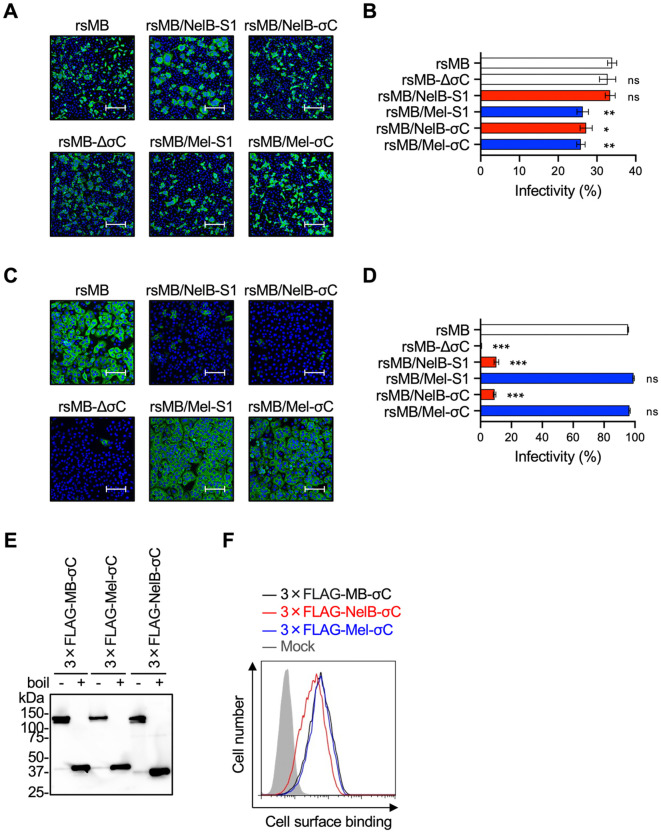
Functional difference in σC between MB and NelB strains in infection and cell attachment. (A, B) Infectivity of S1 monoreassortant or σC chimeric viruses in L929 cells, which were infected with the viruses at MOI of 1 PFU/cell and fixed at 12 h post-infection. Virus infected cells were stained with antiserum against MB strain. Scale bar = 200 μm. (B) Percentage of infected cells were calculated by counting the number of virus infected cells and nuclei. Data represents mean scores with standard deviation. Statistical significance is indicated as ns: not significant, *P < 0.05, **P < 0.01. (C, D) Infectivity of S1 monoreassortant or σC chimeric viruses in A549 cells, which were infected with the viruses at MOI of 2 PFU/cell and fixed at 6 h post infection. Virus-infected cells were stained with antiserum against MB strain. Scale bar = 100 μm. (D) Percentage of infected cells were calculated by counting the number of virus-infected cells and nuclei. Data represents mean scores with standard deviation. Statistical significance is indicated as ns: not significant, ***P < 0.001. (E) Immunoblot analysis of FLAG-tagged recombinant σC proteins. Recombinant proteins were prepared for SDS-PAGE with or without denaturation step. The proteins were detected using an anti-FLAG antibody. (F) Cell surface binding of recombinant σC proteins in A549 cells. The cells were non-enzymatically harvested and mixed with recombinant proteins at 4C for 1 h. Cells were stained with mouse anti-FLAG antibody and Alexa488-conjugated anti-mouse IgG. Cell with recombinant protein was detected using Flow cytometry.

Although several studies have reported mouse models for NBV infection [22,23,25,26], little is known about the target cells in the lungs of infected mice. Therefore, we performed *in vitro* characterization of the mutant viruses. Although rsMB/NelB-S1 and rsMB/NelB-σC express σC protein in the purified virions, they were not virulent in the mouse model (Figs 2B, and 3B and 3E). As σC plays a crucial role in cell attachment and entry, we hypothesized that NelB-σC does not efficiently mediate receptor-dependent entry and infection. We previously identified human lung A549 cells as cell line that NBV MB strain infects in a σC-dependent manner [31]. To further analyze the difference between the effect of σC in the MB and NelB strains during viral infection, we compared the infectivity of the S1 monoreassortant and σC chimeric viruses with that of the wild-type rsMB in the L929 and A549 cells. Consistent with the previous report, rsMB and rsMB-ΔσC showed similar infectivity in L929 cells (Fig 4A and 4B), but rsMB-ΔσC exhibited poor infectivity in A549 cells (Fig 4C and 4D). Furthermore, rsMB/Mel-S1 and σC chimeric viruses (rsMB/NelB-σC and rsMB/Mel-σC) showed slightly lower infectivity in L929 cells compared with that of rsMB (Fig 4A and 4B). In contrast, we observed a distinct difference in viral infectivity in A549 cells. That is, rsMB/NelB-S1 and rsMB/NelB-σC showed substantially low infectivity in A549 cells, whereas rsMB/Mel-S1 and rsMB/Mel-σC infected the A549 cells to a similar extent as that of rsMB (Fig 4C and 4D). These results suggest that the σC protein of the NelB strain differs in its capacity to infect A549 cells compared with that of the MB or Mel strains.

We examined whether the poor infectivity of rsMB/NelB-σC in A549 cells was caused by a defect in the binding activity of NelB σC by performing a cell-surface binding assay using recombinant σC proteins. The prepared Flag-tagged recombinant proteins showed bands between 100 and 150 kDa, corresponding to the size of trimeric σC in the absence of a denaturation step (Fig 4E). Although the peak score was slightly lower than that observed for σC proteins derived from the MB or Mel strains, binding of NelB σC to the surface of A549 cells was observed. (Fig 4F). To further confirm the role of σC on cell attachment using recombinant viruses, we performed a cold-binding assay in A549 and L929 cells followed by q-PCR. The q-PCR results demonstrated that rsMB/NelB-σC bound to the surface of A549 and L929 cells at levels comparable to those of rsMB and rsMB/Mel-σC (S2A and S2B Fig). These results suggest that the difference between the σC proteins of the NBVs isolated from bat and human samples affects virus infectivity only after the cell attachment step, which subsequently affects the virulence of the viruses *in vivo*.

### Functional screening of key amino acids in MB σC for efficient infection in A549 cells

**Fig 5:**
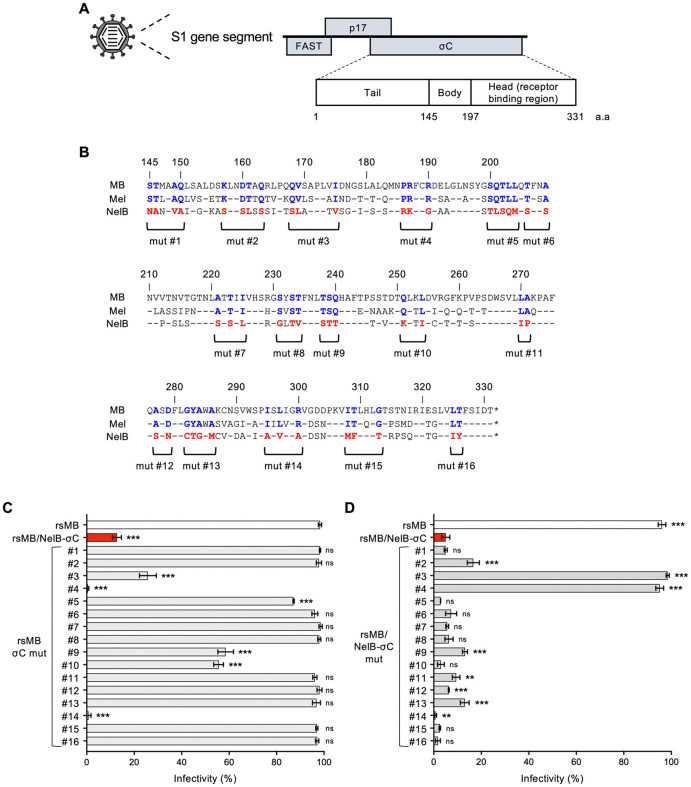
Functional screening of key amino acids in MB σC for efficient infection in A549 cells. (A) Schematic of S1 gene segment and three domains (Tail, body, and head) in σC gene. The numbers indicate amino acid positions in σC protein. (B) Amino acid alignment of σC body and head domains among MB, Mel, and NelB strains. Amino acids conserved between MB and Mel strains but different from those in NelB strain are highlighted in blue or red. (C, D) Infectivity of σC point mutant viruses in A549 cells. The cells were infected with σC point mutant viruses in genetic background of (C) rsMB or (D) rsMB/NelB-σC at MOI of 2 PFU/cell and fixed at 6 h post infection. The cells were stained with antiserum against MB and DAPI. Percentage of infected cells was calculated by counting the number of virus-infected cells and nuclei. Data represent mean scores with standard deviation. Statistical significance is indicated as ns: not significant, **P < 0.01, ***P < 0.001.

The structural protein σC comprises the N-terminal tail, body, and C-terminal head domains (Fig 5A). The C-terminal head domain is required for cell surface binding of MB σC to A549 cells [31]. We further analyzed the functional difference of σC in infectivity by generating a panel of σC mutant viruses. As the amino acid identity of σC proteins varies even among the NBVs isolated from humans (55.6–100%) [8,10,13,15,19,21,22,24], we focused on 48 amino acids in the body or head domains conserved between the MB and Mel strains but differ from those of the NelB strain (Fig 5B). We divided these amino acids into 16 groups with 2–5 amino acids grouped closely together (Table 2). Then, we generated recombinant MB strains with mutations in the 16 sites and compared the infectivity in the A549 cells (Figs 5C and S3). Among the 16 mutant viruses, 5 mutant viruses (rsMB-σC mutant #3, 4, 9, 10, and 14) showed decreased infectivity compared with that of the wild-type rsMB in A549 cells (Fig 5C). Particularly, we observed a pronounced decrease in infectivity of the two mutant viruses rsMB-σC mutant #4 and 14 (Fig 5C). As some mutations may disrupt the conformation of the σC protein, which would result in a loss of infectivity, we generated reciprocal mutant viruses in rsMB/NelB-σC backgrounds (Fig 5B and Table 3) and searched for mutant viruses that infected the A549 cells. We found that the rsMB/NelB-σC mutant #3 and 4 showed similar levels of infectivity as that of rsMB in the A549 cells; however, the other 14 viruses including rsMB/NelB-σC mutant #9, 10, and 14 showed poor infectivity in the A549 cells (Figs 5D and S4). Homology modeling of the σC protein showed that six amino acids introduced in mutant #3 (168Q, 169V, and 175I) and #4 (186P, 187R, and 190R) were located in the body domain of σC (S5 Fig). Based on the results of the virus infectivity assay, we concluded that amino acids in the σC body domain contribute to the different infectivity of MB and NelB strains in the A549 cells.

**Table 2:** Amino acid mutations introduced in MB σC protein of rsMB.

Virus name	Mutations introduced in MB-σC
rsMB-σC-mutant #1	S145N-T146A-A149V-Q150A
rsMB-σC-mutant #2	K157S-D160S-T161L-Q163S
rsMB-σC-mutant #3	Q168S-V169L-I175V
rsMB-σC-mutant #4	P186R-R187K-R190G
rsMB-σC-mutant #5	S200T-Q201L-T202S-L203Q-L204M
rsMB-σC-mutant #6	T206S-A209S
rsMB-σC-mutant #7	A221S-T223S-I225L
rsMB-σC-mutant #8	S231G-S233T-T234V
rsMB-σC-mutant #9	T238S-S239T-Q240T
rsMB-σC-mutant #10	Q251K-L254I
rsMB-σC-mutant #11	L270I-A271P
rsMB-σC-mutant #12	A277S-D279N
rsMB-σC-mutant #13	G282C-Y283T-A284G-A285M
rsMB-σC-mutant #14	I295A-L297V-R300A
rsMB-σC-mutant #15	I308M-T309F-G313T
rsMB-σC-mutant #16	L325I-T326Y

**Table 3:** Amino acid mutations introduced in NelB σC protein of rsMB/NelB-σC.

Virus name	Mutations introduced in NelB-σC
rsMB/NelB-σC-mutant #1	N145S-A146T-V149A-A150Q
rsMB/NelB-σC-mutant #2	S157K-S160D-L161T-S163Q
rsMB/NelB-σC-mutant #3	S168Q-L169V-V175I
rsMB/NelB-σC-mutant #4	R186P-K187R-G190R
rsMB/NelB-σC-mutant #5	T200S-L201Q-S202T-Q203L-M204L
rsMB/NelB-σC-mutant #6	S206T-S209A
rsMB/NelB-σC-mutant #7	S221A-S223T-L225I
rsMB/NelB-σC-mutant #8	G231S-T233S-V234T
rsMB/NelB-σC-mutant #9	S238T-T239S-T240Q
rsMB/NelB-σC-mutant #10	K251Q-I254L
rsMB/NelB-σC-mutant #11	I270L-P271A
rsMB/NelB-σC-mutant #12	S277A-N279D
rsMB/NelB-σC-mutant #13	C282G-T283Y-G284A-M285A
rsMB/NelB-σC-mutant #14	A295I-V297L-A300R
rsMB/NelB-σC-mutant #15	M308I-F309T-T313G
rsMB/NelB-σC-mutant #16	I325L-Y326T

### Virulence of recombinant virus with mutations in the σC body domain in the mouse model

**Fig 6:**
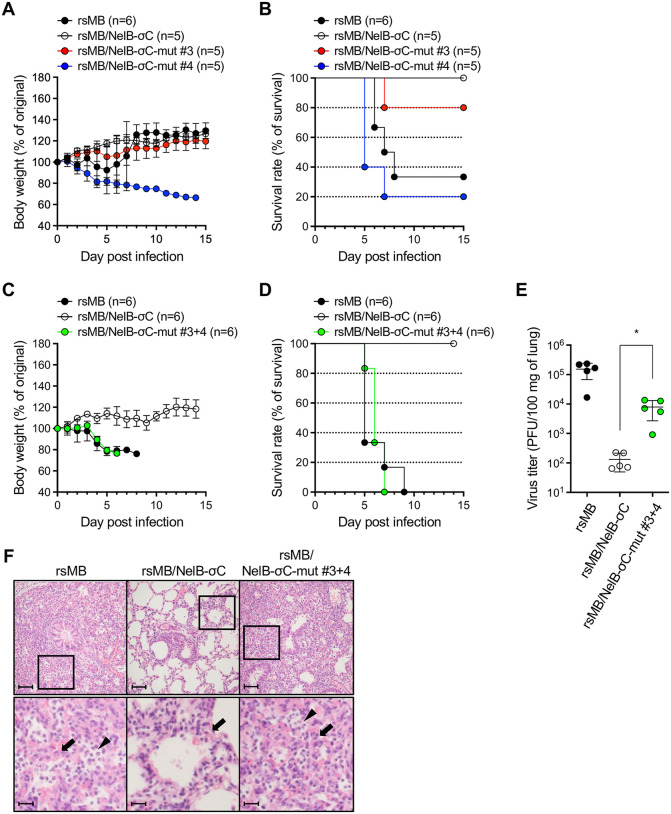
Virulence of recombinant virus with mutations in σC body domain in the mouse model. (A, C) Body weight loss and (B, D) survival curve of mice infected with σC mutant viruses. Four-week-old C3H mice were intranasally inoculated with 2 × 10^5^ PFU/head of (A) rsMB (n = 6), rsMB/NelB-σC (n = 5), rsMB/NelB-σC-mut #3 (n = 5), or rsMB/NelB-σC-mut #4 (n = 5) or (C) rsMB (n = 6), rsMB/NelB-σC (n = 6), or rsMB/NelB-σC-mut #3 + 4 (n = 6) and monitored for two weeks. (A, C) Body weight is expressed as relative value of current weight to original body weight at time of inoculation. Data represent mean scores with standard deviations. (B, D) Percentage of living mice are shown for survival curve. (E) Virus titer in the lung of infected mice. Four-week-old C3H mice were intranasally inoculated with 2 × 10^5^ PFU/head of rsMB (n = 5), rsMB/NelB-σC (n = 5), or rsMB/NelB-σC-mut #3 + 4 (n = 5) and euthanized at 6 days post infection. Virus titers in the lung homogenates were determined using plaque assay. Data represent mean scores with standard deviations. Statistical significance is indicated as *P < 0.05. (F) Histopathological analysis of lungs from mice infected with rsMB, rsMB/NelB-σC, or rsMB/NelB-σC-mut #3 + 4. The top row shows representative lung histopathology images (scale bar = 50 μm). The bottom row shows higher-magnification images corresponding to the boxed regions in the top row. Inflammatory cell infiltration into the alveolar space and alveolar wall is indicated by arrowheads and arrows, respectively (scale bars = 20 μm). Four-week-old C3H mice were intranasally inoculated with 2 × 10^5^ PFU/head of rsMB, rsMB/NelB-σC, or rsMB/NelB-σC-mut #3 + 4 and euthanized at 6 days post inoculation. Lungs were fixed with 10% formalin and stained with hematoxylin and eosin.

Next, we examined whether the mutations in the σC body domain of the NelB strain also affected the virulence of the viruses *in vivo* by intranasally inoculating the C3H mice with rsMB/NelB-σC-mutant #3 or 4 and monitoring the mice for body weight loss and survival. The mice infected with rsMB/NelB-σC-mutant #3 showed mild body weight loss compared with those of the mice infected with rsMB/NelB-σC, and one out of five inoculated mice (20%) died (Fig 6A and 6B). Notably, rsMB/NelB-σC #4 caused severe body weight loss and the mortality of four out of five (80%) inoculated mice (Fig 6A and 6B). The recombinant virus with both mutations in the NelB-σC body domain (rsMB/NelB-σC mutant #3 + 4) caused body weight loss and mortality in all inoculated mice (Fig 6C and 6D). Furthermore, these mutations enhanced viral replication in the lungs of the inoculated mice compared with that observed for rsMB/NelB-σC and resulted in severe lung damage (Fig 6E and 6F).

Next, we attempted to determine the single critical amino acid residue in the body domain that was associated with infectivity and lethal respiratory tract infection *in vivo*. We separately introduced amino acid mutations in rsMB/NelB-σC and generated six single-point mutant viruses (rsMB/NelB-σC-S168Q, -L169V, -V175I, -R186P, -K187R, and -G190R) and compared their infectivity in A549 cells. Three of these viruses (rsMB/NelB-σC-S168Q, -L169V, and -R186P) caused substantial infection in the A549 cells (S6A Fig), which indicated that not all amino acids introduced in the mutant viruses #3 and 4 were required for the efficient infection of the A549 cells. However, all mice inoculated with the three viruses survived without exhibiting body weight loss (S6B and S6C Fig). These results indicate that the three amino acids introduced in either mutant #3 (S168Q, L169V, and V175I) or #4 (R186P, K187R, and G190R) cooperatively contribute to the virulence of the virus *in vivo*.

### Role of amino acids in the σC body domain in σC protein stability

**Fig 7:**
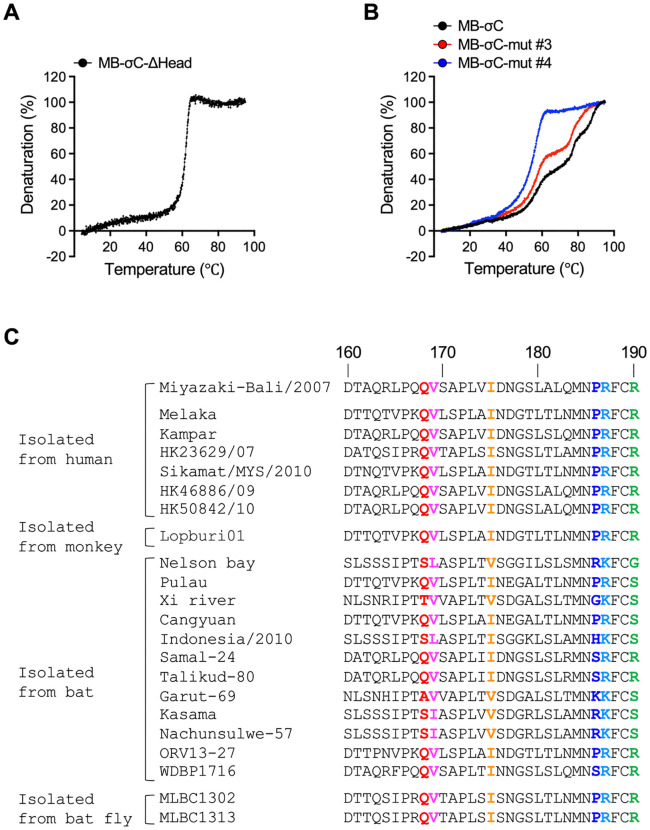
Structural analysis of recombinant σC proteins in the body domain. (A, B) Dissociation curve of recombinant σC proteins. Circular dichroism spectrum of recombinant proteins was measured. Data represent percentage of denaturation. (C) Amino acid alignment of body domain of σC proteins among NBV isolated from human, monkey, bat, and bat fly specimens. Numbers above the alignment show amino acid positions in σC proteins. Six amino acids mutated in rsMB/NelB-σC #3 and rsMB/NelB-σC #4 viruses are highlighted, respectively.

Recombinant NelB-σC bound to the surface of the A549 cells (Fig 4F). The six key amino acids were located in the σC body domain (S5 Fig) and not in the C-terminal head domain, which contains a putative receptor-binding domain (RBD). We attempted to evaluate the role of the amino acids in the NBV σC body domain by assessing the structural stability of the σC protein by comparing the thermal stability of the recombinant σC proteins using circular dichroism (CD) spectroscopy. We used a bacterial protein expression system to prepare the recombinant σC proteins; however, we were unable to obtain a sufficient quantity of NelB-σC. Therefore, we compared the stability of the wild-type MB-σC with that of the MB-σC with mutations introduced in the rsMB-σC mutant #3 or 4. Additionally, we included MB-σC lacking the head domain to consider the role of the head domain in σC stability (MB-σC-ΔHead). The melting curve of MB-σC-ΔHead showed that the protein structure was disrupted at approximately 60 °C (Fig 7A). The melting curve of the wild-type MB-σC showed three distinct transitional phases: below 60 °C, between 60 and 80 °C, and above 80 °C; this was consistent with the three-domain structure of σC (Fig 7B). The difference between MB-σC and MB-σC-ΔHead melting curves suggests that the head domain contributes to the structural stability of MB-σC. Compared with that of the wild-type MB-σC, MB-σC mutant #3 and 4 showed lower thermal stability, although both proteins contained the head domain. MB-σC mutant #3 showed two transitional phases: below 60 °C and between 60 and 80 °C (Fig 7B). In contrast, the melting curve of MB-σC mutant #4 changed notably below 60 °C and reached the plateau phase (Fig 7B). These findings indicate that MB-σC mutant #3 and 4 are structurally less stable than the wild-type MB-σC, which suggests that the difference in amino acids in the σC body domain significantly affected σC protein stability. Moreover, we assessed the importance of amino acids in NBV infection by examining the amino acid sequences among the NBV isolated from humans and bats and constructing a phylogenetic tree. Notably, the amino acids were conserved among all human isolates, although the bat isolates contained different amino acids (Fig 7C). The phylogenetic tree generated from NBV σC amino acid sequences showed that bat isolates were divided into three clusters. One cluster comprised bat isolates, including NelB, Indonesia/2010, Kasama, Nachunsulwe-57, Xi river, and Garut-69, all of which possessed different amino acids at the identified positions. In contrast, bat isolates in the other two clusters exhibited amino acids similar to those of the MB strain (S7 Fig). This suggests that these amino acids may play an essential role in the transmission to and infection of humans.

## Discussion

In this study, we showed the differences in virulence phenotypes between the NelB strain and a human isolate using a mouse model for acute respiratory tract infection (Fig 1A–1D). We identified that the S1 gene segment, which is the most divergent gene segment in the NBV genome, is associated with NBV virulence (Fig 2A–2D). After the isolation of the Melaka strain from a human patient, several NBV strains have been isolated to date from human, bat, monkey, and bat fly samples [9–24]. However, whether the gene segment(s) or protein(s) of the NBV are involved in its virulence remains unclear. We showed that the cell attachment protein σC determines the strain-specific difference in NBV pathogenesis (Fig 3B, 3C, and 3G). Additionally, we found that the S1 gene segment in a human isolate is a determinant of NBV pathogenesis; however, whether the other nine gene segments contribute to the difference in NBV virulence remains unclear. Moreover, the other steps in addition to the entry step contribute to strain-specific differences in viral replication. Several viruses such as influenza virus NS1, flavivirus NS5, and rotavirus NSP1 contain interferon antagonists [32,33]. Additionally, the mammalian orthoreovirus, a well-studied model virus in the genus Orthoreovirus, family *Spinareoviridae*, also inhibits the IFN induction by the core protein μ2 [34]. The difference in the capacity to disrupt interferon signaling results in a difference in viral replication within host cells. Similarly, influenza virus PB2, which is a subunit of the heterotrimeric polymerase, is involved in the species-specific difference in viral replication [35]. A mutation in the avian influenza virus PB2 increases its polymerase activity in mammalian cells by facilitating host ANP32 family proteins [36,37]. Therefore, examining the virulence of monoreassortant viruses containing a segment other than the S1 gene segment may help determine whether other gene segments are involved in the different NBV virulence phenotypes. Future studies are required to address the role of other NBV proteins in producing strain-specific differences in viral replication.

The results of the current study indicate that the strain-specific difference of NBV σC may be attributed to the post-attachment step. The variations in amino acids within the cell attachment protein contribute to an increased binding affinity for the cellular receptor. For example, mutations in the SARS-CoV-2 spike protein enhanced the infectivity of the virus. However, in addition to the mutations in the RBD of the spike protein, which directly affected the binding affinity of the spike to the receptor ACE2, mutations outside the RBD of the spike protein increased viral infectivity by altering the structure of the spike protein and enhancing the efficacy of proteolysis of the spike protein [38]. In the present study, we performed a cell-surface binding assay to determine whether the poor infectivity of rsMB/NelB-σC may be attributed to the attachment or post-attachment steps. We found that NelB-σC bound to the surface of A549 cells (Fig 4F). In a previous study, we showed that recombinant MB-σC protein lacking the head domain did not bind to the cell surface of A549 cells [31], which suggests that the head domain is required for cell surface binding in A549 cells. Therefore, the result of the binding assay using NelB-σC indicates that the poor infectivity of rsMB/NelB-σC is caused by the post-attachment step. Furthermore, the result suggests that the RBD of σC is probably conserved in the NelB strain, although the σC proteins are divergent among NBV strains. Recently, heparan sulfate and cytokeratin 1 were reported to be involved in cell surface attachment [39,40]. Human and bat lung cells contain heparan sulfates with different sulfation levels [41]. Thus, comparing the binding affinity of the σC proteins from human and bat NBV isolates to heparan sulfate from human and bat cells would help examine the role of heparan sulfate in NBV infection.

Moreover, we generated a panel of mutant viruses with mutations in the body or head domains of σC (Figs 5A and S5 and Tables 2 and 3) and found that the mutations in σC body domain played a vital role in the infectivity of the virus in A549 cells (Figs 5C and 5D; S3 and S4) and virulence *in vivo* (Fig 6A–6F). Furthermore, we found that the key amino acids in MB σC (168Q, 169V, 175I, 186P, 187R, and 190R) contribute to the structural stability of σC (Fig 7A and 7B). In the case of the mammalian reovirus, the viruses enter the cells via receptor-mediated endocytosis, where low pH and endocytic protease trigger the uncoating of the outer capsid protein and conformational change in the spike protein σ1 [42]. Mutations in the σ1 body domain yielded different results depending on the mutated site. Some mutations in the σ1 body domain decreased not only cell attachment, but also virus replication by affecting a post-disassembly step [43,44]. The amino acids identified in this study may increase the efficacy of the virion uncoating process in the endosome during the entry process. The role of the body domain in the entry process needs to be examined in further studies.

Amino acid alignment showed that mutations of the amino acids in the body domain are not conserved among the bat NBV isolates. However, these amino acids are conserved among the human NBV isolates, which suggests possible selective pressure on human isolates (Fig 7C). Although the NBV σC from the human isolates plays a vital role in infectivity in A549 cells and virulence *in vivo*, we previously showed that NBV infects several cell lines (e.g., BHK-21, CHO-K1, L929, and Vero cells) in a σC-independent manner [31]. Currently, the role of σC-independent infection *in vivo* remains unclear; however, if NBV propagates *in vivo* via a σC-independent mechanism, the selective pressure on σC may decrease and lead to the accumulation of divergent amino acids in σC. Hence, the replication of the σC knock-out virus *in vivo* needs to be analyzed to validate this hypothesis.

Currently, the virulence of two NBV strains isolated from bats (Samal-24 and Nachunsulwe-57) has been compared with that of the MB strain in a mouse model [22,25]. Samal-24 strain showed a similar lethal dose 50 (4.2 × 10^3^ PFU/head) as that of the MB strain (6.8 × 10^3^ PFU/head) [25]. The Nachunsulwe-57 strain was less virulent than the MB strain [22]. Among the six amino acids in the σC body domain related to virulence, five amino acids (with the exception of 186P) of the Samal-24 strain were the same as those in the MB strain (Fig 7C). In contrast, Nachunsulwe-57 strain showed different amino acids than those in the MB strain, and four out of the six amino acids (168S, 175V, 186R, and 187K) were identical to those in the NelB strain (Fig 7C). Therefore, the difference in amino acids in the body domain may explain the varying virulence of other NBV strains. Notably, one bat isolates, ORV13–27 contained the same amino acids at the identified positions as those observed in the human isolates (Figs 7C and S7). Interestingly, NBV isolates obtained from monkey fecal samples (Lopburi01) and bat flies (MLBC1302 and MLBC1313) also possessed the same amino acids at these positions (Figs 7C and S7). If efficient infection and virulent phenotypes are detected for these viruses in the mouse model, it might validate the hypothesis that bats are the source of NBV strains and that some of the NBV strains are capable of being transmitted to humans. Taken together, we demonstrated that the NBV cell attachment protein σC functions as a key factor for both infection and virulence.

## Materials and methods

### Cells and viruses

Murine fibroblast L929 cells, monkey kidney epithelial Vero cells, and human lung carcinoma A549 cells were cultured in Dulbecco modified Eagle medium (DMEM; Nacalai Tesque) supplemented with 5% fetal bovine serum (FBS; Gibco), 100 I.U./ml penicillin, and 100 μg/ml streptomycin (Nacalai Tesque). Baby hamster kidney (BHK) cells expressing T7 RNA polymerase (BHK/T7-9) were provided by Dr. Naoto Ito [45]. They were cultured in DMEM containing 5% FBS, 100 I.U./ml penicillin, and 100 μg/ml streptomycin, and 600 μg/ml hygromycin was included during every other passage. NBV NelB strain was provided by Dr. Richard W. Compans [6]. Wild-type and recombinant viruses were propagated in L929 cells or Vero cells as described previously [31].

### Plasmid construction

The rescue plasmids for the MB strain and the S1 gene segment of the Mel strain were constructed as described previously [31]. To construct the rescue plasmid of the S1 gene segment of the Indonesia/2010 strain, its sequence (Accession number: KM279386) was synthesized (Eurofins Genomics) and used to replace the S1 gene in pT7-MB-S1. To construct the rescue plasmids for chimeric viruses between MB and NelB strains (pT7-MB-S1-NelB-FAST, pT7-MB-S1-NelB-p17, or pT7-MB-S1-NelB-σC), nucleotide sequences encoding FAST (27–314 nt), p17 (277–705 nt), or σC (572–1567 nt) in pT7-MB-S1 was replaced with those from the NelB strain, respectively. The rescue plasmids for other σC chimeric viruses (pT7-MB-S1-Mel-σC and pT7-MB-S1-Idn-σC) were generated by replacing the nucleotide for MB-σC with those for Melaka-σC or Indonesia/2010-σC in pT7-MB-S1, respectively. To construct the rescue plasmids for σC point mutant viruses, pT7-MB-S1 or pT7-MB-S1-NelB-σC was used as templates, and mutations were introduced by performing site-directed PCR. The lists of the constructed plasmids are presented in Tables 2 and 3. The three copies of FLAG (3 × FLAG)-tagged σC protein expression vector for mammalian cells (p3 × FLAG-MB-σC) was generated previously [31]. Other 3 × FLAG-tagged σC protein expression vectors (p3 × FLAG-Mel-σC and p3 × FLAG-NelB-σC) were generated by replacing the MB σC ORF with those for the Mel or NelB strains. The protein expression vector for bacterial cells (pTrcHisA-MB-σC) was generated previously [31]. To construct the protein expression vector lacking the MB-σC head domain, the nucleotide sequence encoding the σC head domain (197–331 a. a.) was deleted using inverse PCR with pTrcHisA-MB-σC as the template. To construct the protein expression vector of MB-σC with mutations in the body domain positions #3 and 4, nucleotide mutations were introduced using inverse PCR with pTrcHisA-MB-σC as the template. To construct the protein expression vector of NelB-σC, the ORF encoding the σC of the NelB strain was cloned into pTrcHisA vector (Life Technologies). All plasmids were sequenced through Sanger sequencing before use.

### Reverse genetics

Recombinant NBV strains were generated using plasmid-based reverse genetics as described previously [31]. Briefly, a monolayer of BHK/T7-9 cells was transfected with the mixture of 10 rescue plasmids (0.66 μg/plasmid) using 2 μl/μg of plasmid of TransIT-LT1 (Mirus). The cells were cultured for up to 5 days and lysed by performing three rounds of freezing and thawing. Then, the rescued viruses were passaged in the L929 cells. For σC point mutant viruses, the nucleotide sequence of the σC gene was confirmed using Sanger sequencing.

### Antibodies

The following monoclonal antibodies were used in this study: anti-FLAG (clone M2, Sigma-Aldrich) and anti-β-actin (clone AC-15, Sigma-Aldrich). Mouse antiserum against MB strain was generated previously [31]. Rabbit antiserum against MB σB was generated previously [46]. To generate mouse antiserum against NelB-σC, recombinant NelB-σC protein with an N-terminal poly-histidine tag was expressed in BL21 by transforming pTrcHisA-NelB-σC, which was purified using the cOmplete His-Tag Purification Resin (Roche). The purified protein was mixed with Alhydrogel Adjuvant 2% (InvivoGen) and injected into 4-week-old ICR mice (Clea Japan). The mice were boosted twice with the protein–adjuvant mixture, and the sera were collected from the mice.

### Plaque assay

A monolayer of L929 cells seeded on a 12- or 24-well plate was infected with a 10-fold serially diluted virus stock and incubated at 37 °C for 1 h, after which the inoculum was removed, and DMEM containing 0.8% SeaPlaque (Lonza) was overlaid. The cells were cultured for 3–5 days until the plaque became visible. The cells were fixed with 4% paraformaldehyde and stained with 0.05% crystal violet solution. Viral titer was expressed as plaque-forming units per ml (PFU/ml).

### Focus-forming unit assay

A monolayer of L929 cells was infected with 10-fold serially diluted viruses and incubated at 37 °C for 1 h. After adsorption, the inoculum was removed, and the medium was added, followed by culturing the cells overnight. The cells were fixed with 4% paraformaldehyde and permeabilized with PBS containing 0.05% Tween-20. The cells were stained with mouse antiserum against the NBV MB strain and CF488-conjugated secondary anti-mouse IgG antibody (Biotium). The number of foci was counted using a FluoView FV1000 laser scanning confocal microscope (Olympus). Viral titer was expressed as focus-forming units per ml (FFU/ml).

### Mouse experiment

Four-week-old C3H strain mice were purchased from Clea Japan. The mice were intranasally inoculated with 20 μl of diluted viruses (2 × 10^5^ PFU or 2 × 10^6^ PFU). The mice were monitored for changes in body weight and survival for 14 days. To determine the viral titer in the lungs, 4-week-old C3H mice were intranasally inoculated with the viruses, and the lungs were harvested at 6 days post-inoculation. The lungs were mixed with DMEM and homogenized with Beads Smash 12 (Wakenyaku). After centrifugation, the supernatant was used for virus titration through plaque assay. Viral titer was expressed as PFU/100 mg of lung. For histopathology, 4-week-old C3H mice were intranasally inoculated with the viruses, and the lungs were harvested at 6 days post-inoculation, fixed in 10% formalin, and processed routinely to create paraffin-embedded tissue sections. Histopathological changes were examined using hematoxylin and eosin staining. The animal experiment protocol was approved by the Research Institute for Microbial Diseases, the University of Osaka (Approval number: Bi-Dou-25-04-0).

### Preparation of FLAG-tagged recombinant σC proteins

FLAG-tagged σC proteins were prepared using 293T cells as described previously [31]. Briefly, 293T cells were transfected with p3 × FLAG-MB-σC, p3 × FLAG-Mel-σC, or p3 × FLAG-NelB-σC using 1 mg/ml of polyethyleneimine solution (Cosmo Bio). After 48 h of incubation, the recombinant proteins expressed in the cells were collected using ANTI-FLAG M2 Affinity Gel (Sigma-Aldrich) after cell lysis using a buffer (50 mM Tris-HCl [pH 7.4], 150 mM NaCl, and 1% Triton X-100). The recombinant proteins were competitively eluted using 3 × FLAG peptide. The peptide was removed through dialysis using a Vivaspin 6 (Sartorius). To confirm the trimer formation of the recombinant proteins, the FLAG-tagged recombinant σC proteins were resolved on e-PAGEL 5–20% gradient gel (ATTO) with or without a denaturing step by boiling at 95 °C for 5 min. The samples were transferred onto a PVDF membrane (Millipore), and the recombinant proteins were detected using anti-FLAG antibody (clone M2).

### Virion purification

To prepare purified rsMB/NelB-S1, rsMB/NelB-σC, and rsMB-ΔσC virions, L929 cells were infected with these viruses. The cells were lysed by performing freeze and thaw, and the viruses were pelleted from the cell lysate through centrifugation. Next, the obtained virions were purified using CsCl density-gradient centrifugation.

### Immunoblotting

Purified virion and cell lysate samples were mixed with 2 × Laemmli sample buffer, boiled at 95 °C for 5 min. Samples were resolved on e-PAGEL 5–20% gradient gel, and transferred onto PVDF membrane. The viral proteins and control β-actin were detected using the appropriate antibodies.

### Cell fusion formation assay

Vero cells were infected with the viruses at a multiplicity of infection (MOI) of 0.1 PFU/cell and cultured for 12 h. The cells were fixed with 4% paraformaldehyde and permeabilized with PBS containing 0.05% Tween-20. Viral protein was stained with mouse antiserum against MB, followed by staining with CF488-conjugated anti-mouse IgG. The nuclei were stained with 4’,6-diamidino-2-phenylindole (DAPI).

### Cell infectivity assay

L929 or A549 cells were infected with the viruses at an MOI of 1 or 2 FFU/cell, respectively. Next, the L929 and A549 cells were fixed with 4% paraformaldehyde at 12 h post-infection for L929 cells and 6 h post-infection for A549 cells. Viral antigens were stained with mouse antiserum against the MB strain, followed by staining with CF488-conjugated anti-mouse IgG. The nuclei were stained with DAPI. Images were acquired using a FluoView FV1000 laser scanning confocal microscope (Olympus). The number of viral antigen-positive cells and nuclei were counted and used to calculate the percentage of infection.

### Cell surface binding assay

The cell surface binding capacity of the recombinant σC proteins was analyzed as described previously [31]. Briefly, A549 cells were incubated with FLAG-tagged recombinant proteins at 4 °C for 1 h. Then, the cells were first stained with mouse anti-FLAG M2 antibody and next with CF488-conjugated anti-mouse IgG. The live cells were stained with 10 μg/ml of propidium iodide solution (Sigma Aldrich). The number of cells containing the recombinant protein was analyzed using FACSCalibur (Becton Dickinson). The data were analyzed using FlowJo software.

### Cell surface attachment of recombinant viruses

A549 and L929 cells were seeded into 48-well plates and cultured overnight at 37 °C. The plates were then incubated at 4°C for 1 h, before infection with rsMB, rsMB/NelB-σC, or rsMB/Mel-σC for 1 h at 4°C. After viral adsorption, the cells were washed with cold PBS, and total RNA was extracted using the RNeasy Mini kit (QIAGEN). Reverse transcription was performed using ReverTra Ace (TOYOBO) with random hexamer primers. The resulting cDNA was subjected to q-PCR using primers specific for the L1 gene segment as previously described [29] and GAPDH. The following primers were used for GAPDH: forward primer, 5’-ACCCAGAAGACTGTGGAT-3’; reverse primer, 5’-AGGCCATGCCAGTGAGC-3’. The relative amount of viral RNA was calculated against that for the rsMB-infected samples.

### Homology modeling

Homology modeling of MB σC was performed using Modeller v9.17 [47]. The amino acid sequence of MB σC was obtained from NCBI (accession number: AB521793.1), and the crystal structure of the avian reovirus σC protein (residues 117–326; PDB code: 2jjl) [48] was used as the template. Six amino acid residues in the body domain (Q168, V169, I175, P186, R187, and R190) were rendered as sphere models. The figure was prepared using ChimeraX (ref https://doi.org/10.1002/pro.3943).

### Analysis of structural stability of σC protein

The structural stability of the σC proteins was analyzed using a spectropolarimeter (J-720W JASCO Corporation). Recombinant σC proteins were produced in bacterial BL21 cells and purified using cOmplete His-Tag Purification Resin (Roche), followed by dilution to 0.1 mg/ml with 20 mM phosphate buffer [pH 7.4]. The circular dichroism (CD) ellipticity at 222 nm was monitored over a temperature range of 4–95 °C to assess the thermal denaturation (unfolding) of the recombinant proteins. Thermal denaturation profiles were generated by plotting the temperature-dependent changes in CD ellipticity. The fraction of denatured protein at each temperature was calculated by normalizing the ellipticity values with respect to those obtained at 4 °C (fully folded state) and 95 °C (fully denatured state).

### Amino acid alignment of σC proteins

To compare the amino acid sequence of σC proteins, the nucleotide sequences of the following NBV strains were used: Miyazaki-Bali/2007 (Accession number, AB908284), Melaka (EF026043), Kampar (EU448334), Sikamat/MYS/2010 (JF811580), HK46886/09 (AEP17496), HK50842/10 (AEP17499), Pulau (AY357730), Xi river (GU188274), Cangyuan (NC_025806), Indonesia/2010 (KM279386), Samal-24 (LC110198), Talikud-80 (LC110248), Garut-69 (LC389084), Lopburi01 (KY751019), Kasama (MT505318), Nachunsulwe-57 (LC619335), ORV13–27 (LC632078), WDBP1716 (OP913262), MLBC1302 (OP913242), and MLBC1313 (OP913252). The amino acid alignment of the σC body domain was generated based on these sequences. For phylogenetic analysis, the σC amino acid sequences were aligned using MUSCLE in MEGA 12 and then analyzed using the maximum likelihood method with 1000 bootstrap replicates [49].

### Statistical analysis

Statistical analysis was performed using GraphPad Prism 10 (GraphPad Software, Inc.). Unpaired-t test was used for analyzing the data presented in Figs 1C and 6E. One-way ANOVA was used for analyzing the data presented in Figs 2A, 3A and 3F, 4B and 4D, and 5C and 5D, and S2A and S2B.

## Supporting information

S1 Fig Schematic of S1 gene segments of chimeric viruses harboring FAST, p17, or σC from NelB in MB backbone. Boxes show open reading frames (ORFs) of FAST, p17, and σC. Numbers indicate nucleotide position in the S1 gene segment. Genes from MB and NelB strains are indicated in blue and red, respectively.
(TIF)

S2 Fig Cell attachment of σC chimeric viruses in A549 and L929 cells. (A) A549 cells and (B) L929 cells were infected with rsMB, rsMB/NelB-σC, or rsMB/Mel-σC at 4°C for 1 h. The number of virions bound to the cell surface was quantified by q-PCR. The amount of viral genomic RNA was expressed relative to that of rsMB. Statistical significance is indicated as ns: not significant.
(TIF)

S3 Fig Infectivity of σC point mutant viruses of rsMB in A549 cells. Cells were infected with each virus at MOI of 2 PFU/cell and fixed at 6 h post-infection. Cells were stained with antiserum against MB strain and DAPI. Scale bars = 100 μm.
(TIF)

S4 Fig Infectivity of σC point mutant viruses of rsMB/NelB-σC in A549 cells. Cells were infected with each virus at MOI of 2 PFU/cell and fixed at 6 h post-infection. Cells were stained with antiserum against MB strain and DAPI. Scale bars = 100 μm.
(TIF)

S5 Fig Homology model of MB σC. Structural model of MB σC was generated using crystal structure of avian reovirus σC (117–326 a. a.) as the template. Mutations introduced in mutant #3 and 4 are highlighted in the model.
(TIF)

S6 Fig Infectivity and virulence of selected σC single point mutant viruses. (A) A549 cells were infected with viruses at MOI of 2 PFU/cell, fixed at 6 h post-infection, and stained with antiserum against MB and DAPI. Scale bars = 100 μm. (B, C) Virulence of σC mutant viruses. Four-week-old C3H mice were intranasally inoculated with 2 × 10^5^ PFU/head of rsMB (n = 6), rsMB/NelB-σC (n = 5), rsMB/NelB-σC-S168Q (n = 5), rsMB/NelB-σC-L169V (n = 5), or rsMB/NelB-σC-R186P (n = 5) and monitored for two weeks. (B) Body weight is expressed as relative value of current weight to the original body weight at time of inoculation. Data represent mean scores with standard deviations. (C) Percentage of living mice are shown for survival curve.
(TIF)

S7 Fig Phylogenetic tree of NBV strains based on σC protein sequences. The amino acid sequences of NBV strains were used to generate the phylogenetic tree. The sequences were aligned using MUSCLE and analyzed using the maximum likelihood method with 1000 bootstrap replicates. NBV strains isolated from humans, bats, monkeys, and bat flies were highlighted in red, blue, yellow, and green, respectively. The numbers shown to the right of each strain name indicate the number of amino acids identical to those of the MB strain at the identified positions within the σC body domain.
(TIF)

S1 Data Raw data used for figure generation. (XLSX)
