# Supplementary figures and images for: Nelson Bay Orthoreovirus cell attachment protein σC determines strain-specific differences in infectivity and pathogenesis
Source: PLoS Pathog. 2026 Aug 3;22(8):e1014409. doi: 10.1371/journal.ppat.1014409 (PMC13432128; doi:10.1371/journal.ppat.1014409)

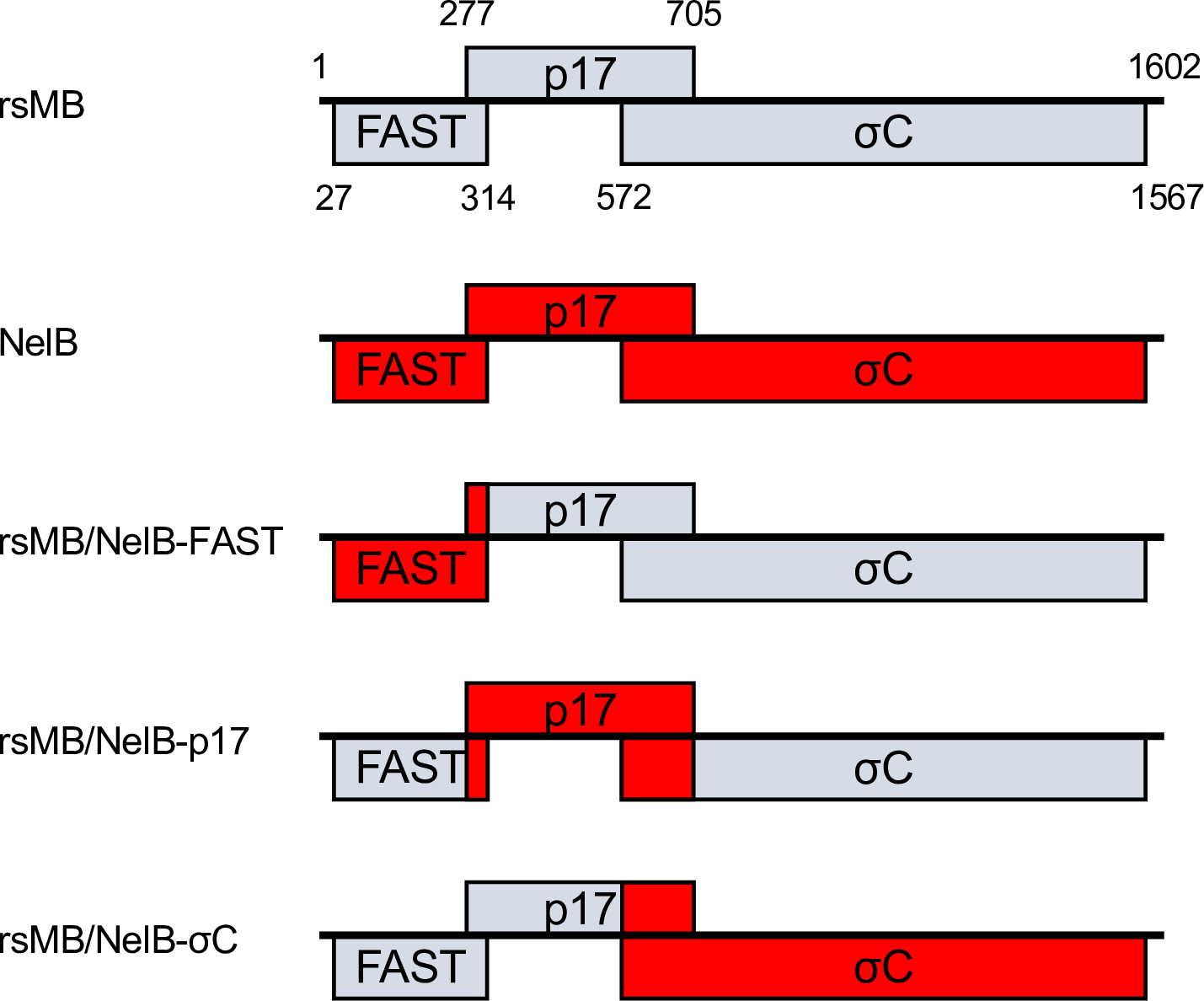

Supplement: S1 Fig — Boxes show open reading frames (ORFs) of FAST, p17, and σC. Numbers indicate nucleotide position in the S1 gene segment. Genes from MB and NelB strains are indicated in blue and red, respectively. (TIF) [file ppat.1014409.s001.tif]

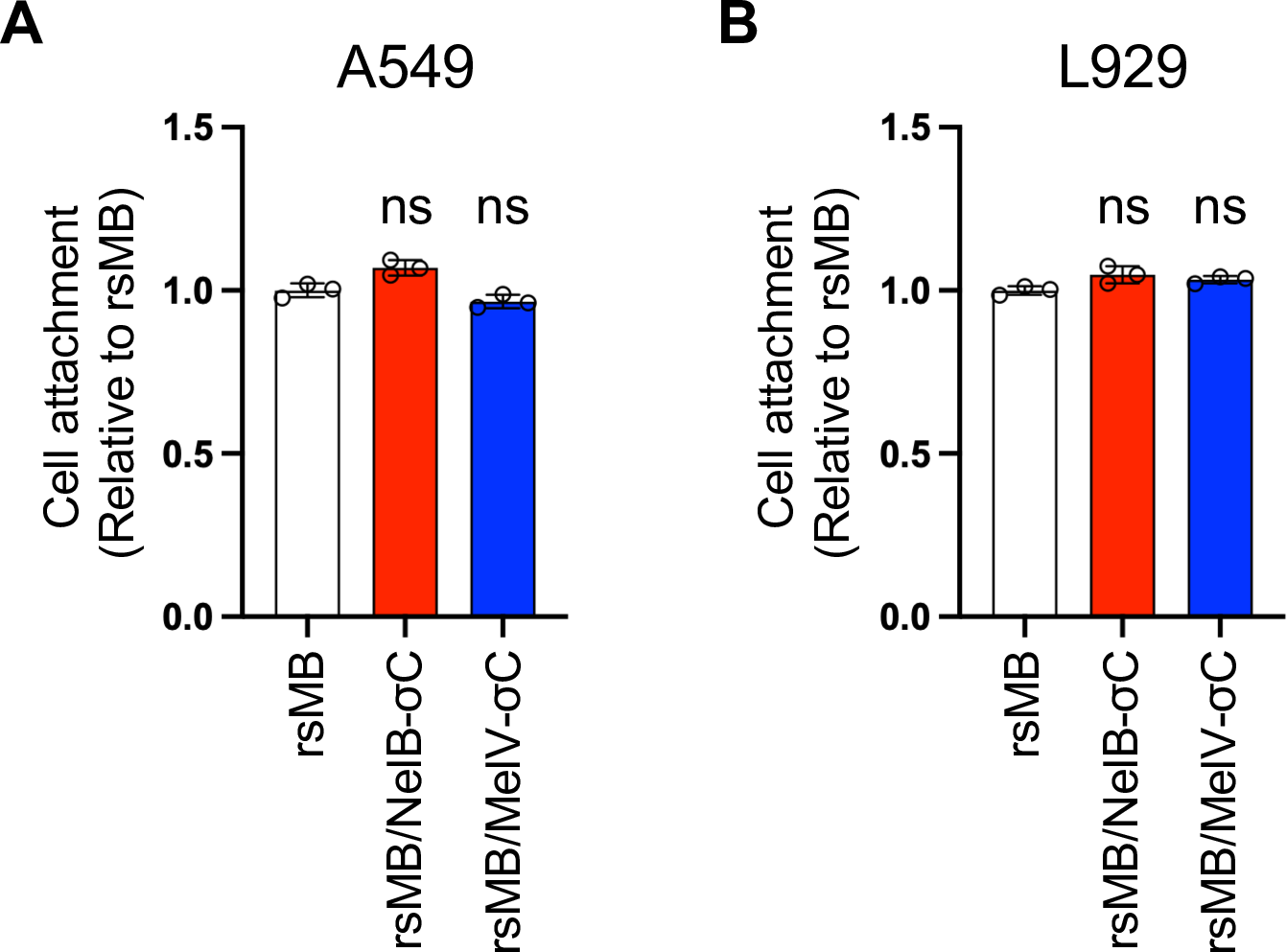

Supplement: S2 Fig — (A) A549 cells and (B) L929 cells were infected with rsMB, rsMB/NelB-σC, or rsMB/Mel-σC at 4°C for 1 h. The number of virions bound to the cell surface was quantified by q-PCR. The amount of viral genomic RNA was expressed relative to that of rsMB. Statistical significance is indicated as ns: not significant. (TIF) [file ppat.1014409.s002.tif]

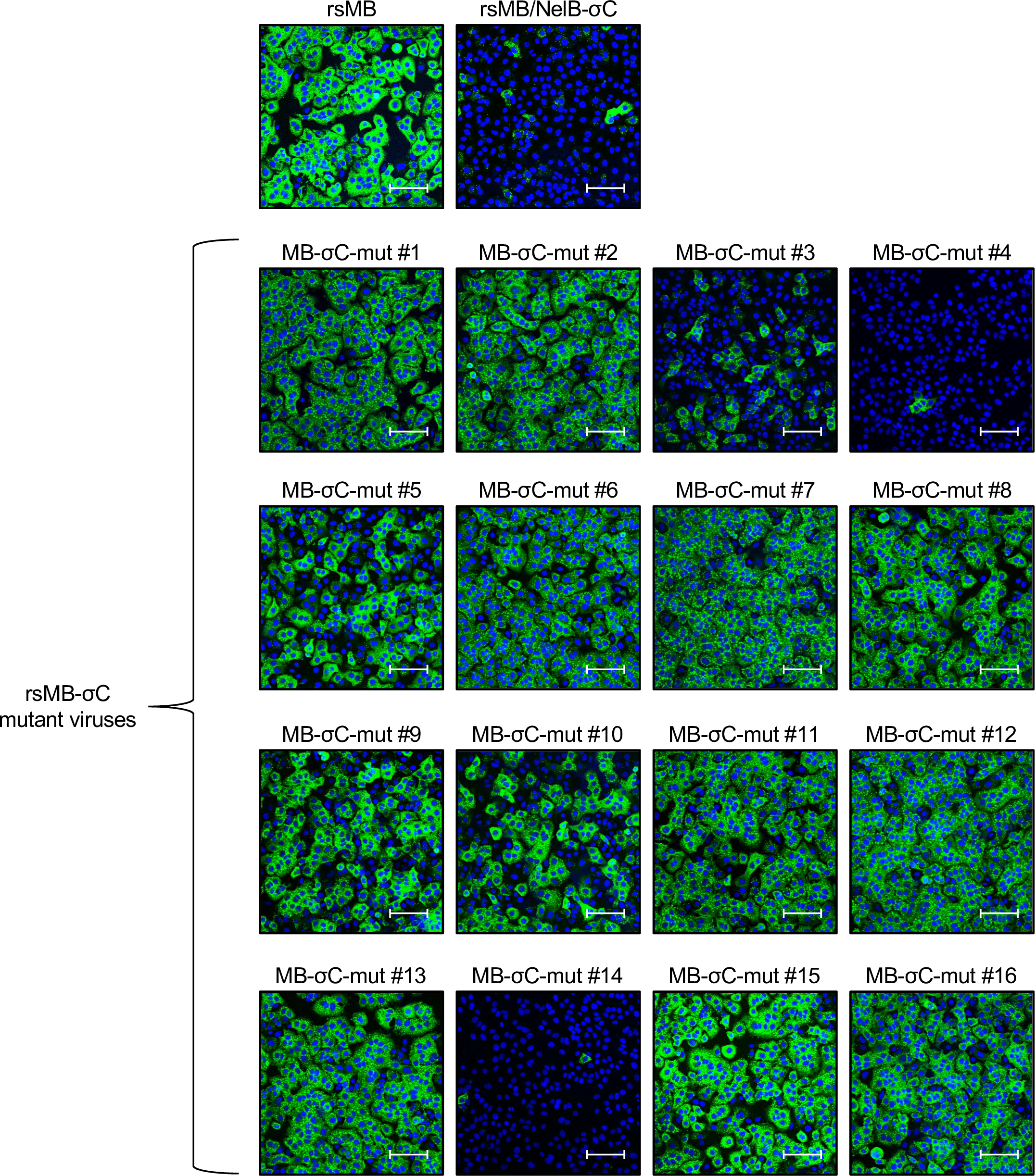

Supplement: S3 Fig — Cells were infected with each virus at MOI of 2 PFU/cell and fixed at 6 h post-infection. Cells were stained with antiserum against MB strain and DAPI. Scale bars = 100 μm. (TIF) [file ppat.1014409.s003.tif]

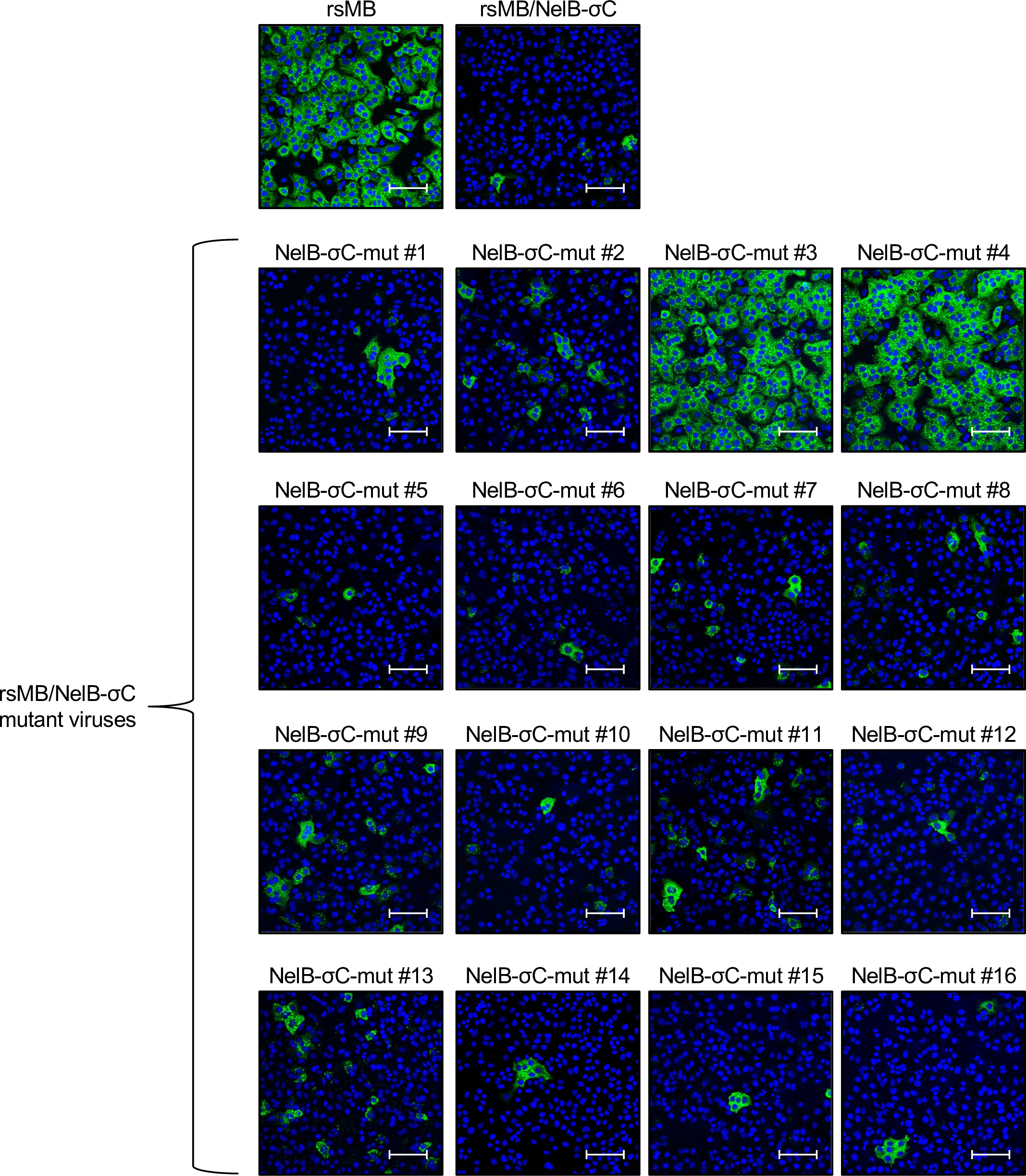

Supplement: S4 Fig — Cells were infected with each virus at MOI of 2 PFU/cell and fixed at 6 h post-infection. Cells were stained with antiserum against MB strain and DAPI. Scale bars = 100 μm. (TIF) [file ppat.1014409.s004.tif]

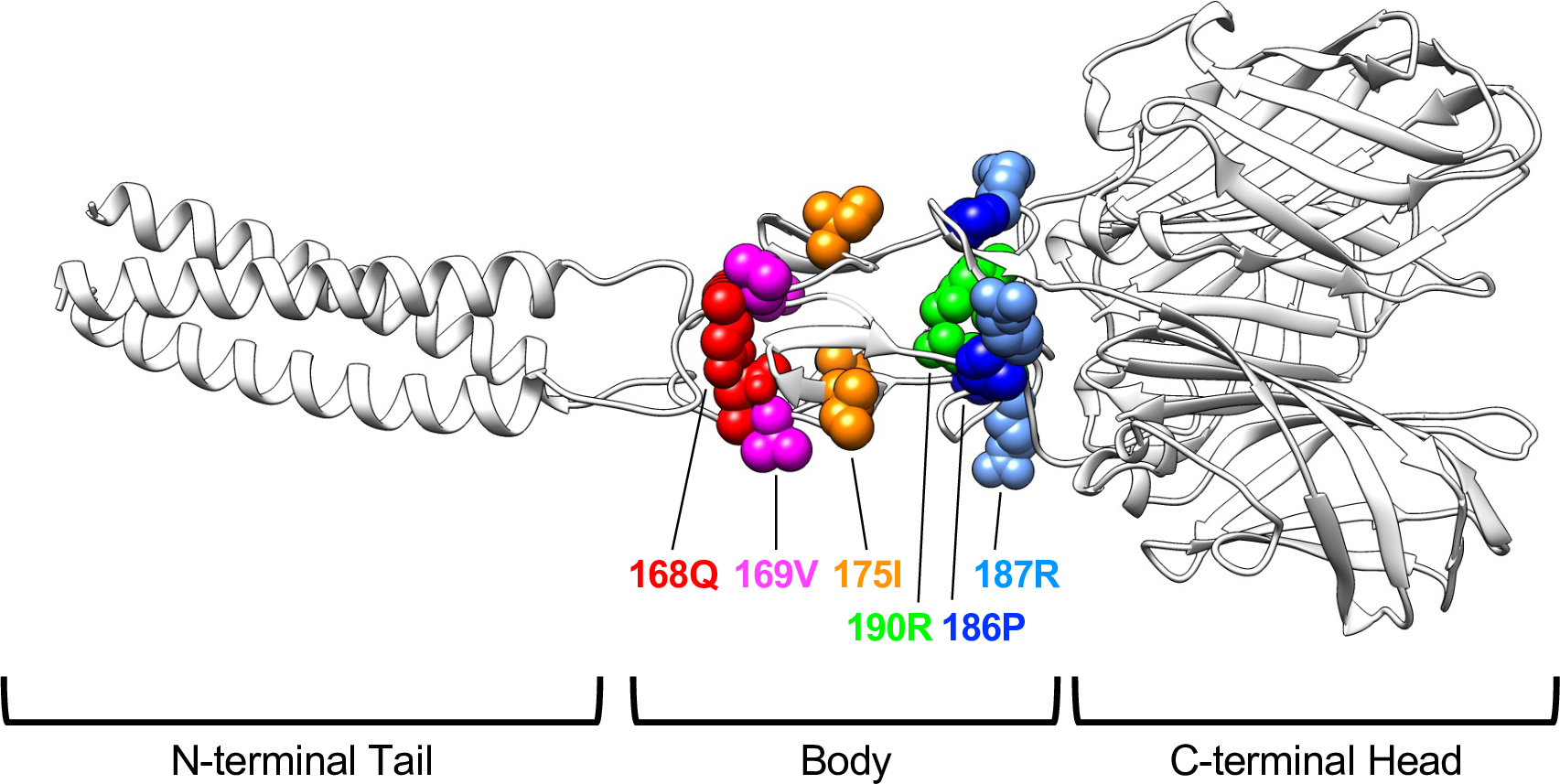

Supplement: S5 Fig — Structural model of MB σC was generated using crystal structure of avian reovirus σC (117–326 a. a.) as the template. Mutations introduced in mutant #3 and 4 are highlighted in the model. (TIF) [file ppat.1014409.s005.tif]

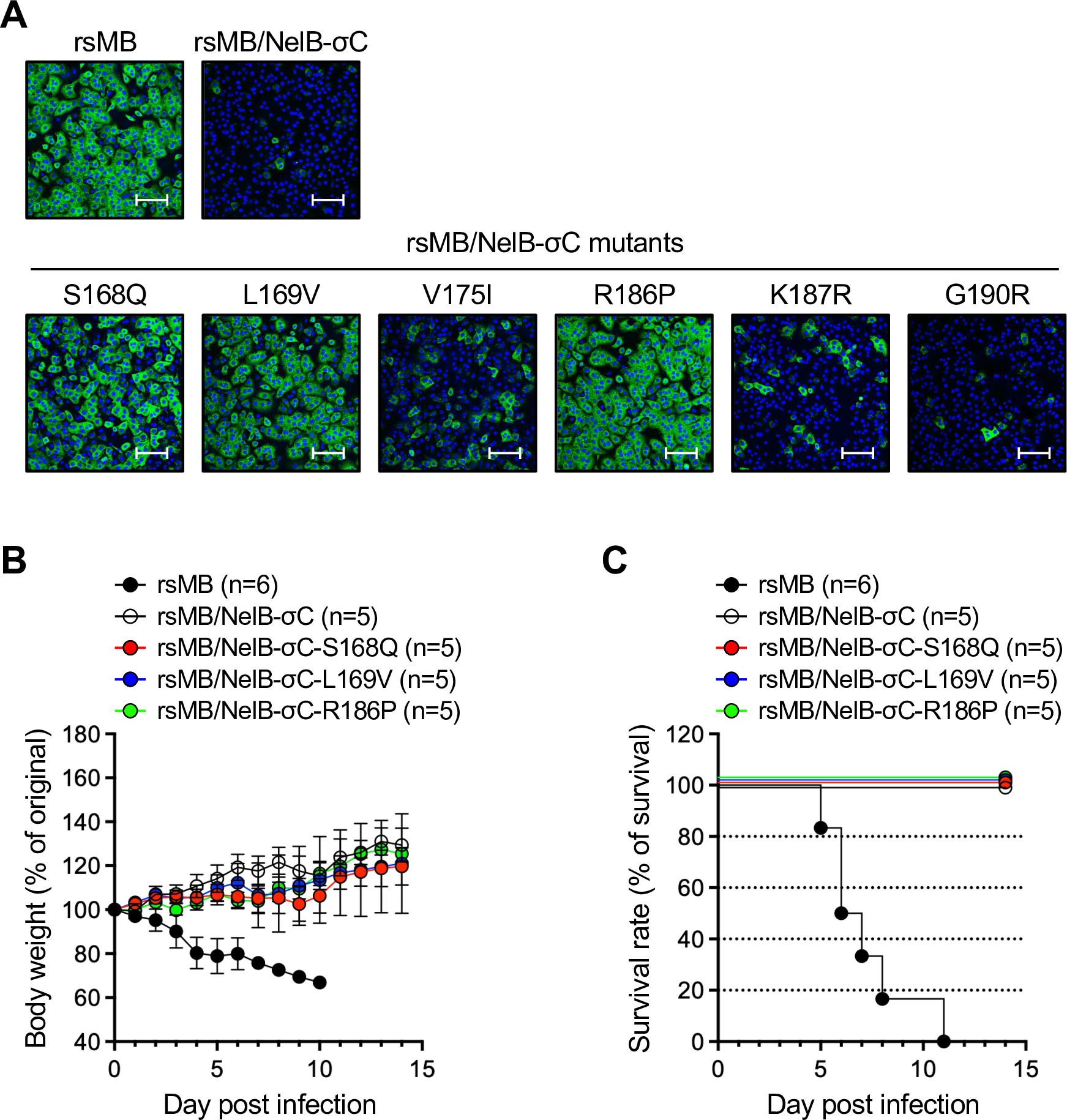

Supplement: S6 Fig — (A) A549 cells were infected with viruses at MOI of 2 PFU/cell, fixed at 6 h post-infection, and stained with antiserum against MB and DAPI. Scale bars = 100 μm. (B, C) Virulence of σC mutant viruses. Four-week-old C3H mice were intranasally inoculated with 2 × 10^5^ PFU/head of rsMB (n = 6), rsMB/NelB-σC (n = 5), rsMB/NelB-σC-S168Q (n = 5), rsMB/NelB-σC-L169V (n = 5), or rsMB/NelB-σC-R186P (n = 5) and monitored for two weeks. (B) Body weight is expressed as relative value of current weight to the original body weight at time of inoculation. Data represent mean scores with standard deviations. (C) Percentage of living mice are shown for survival curve. (TIF) [file ppat.1014409.s006.tif]

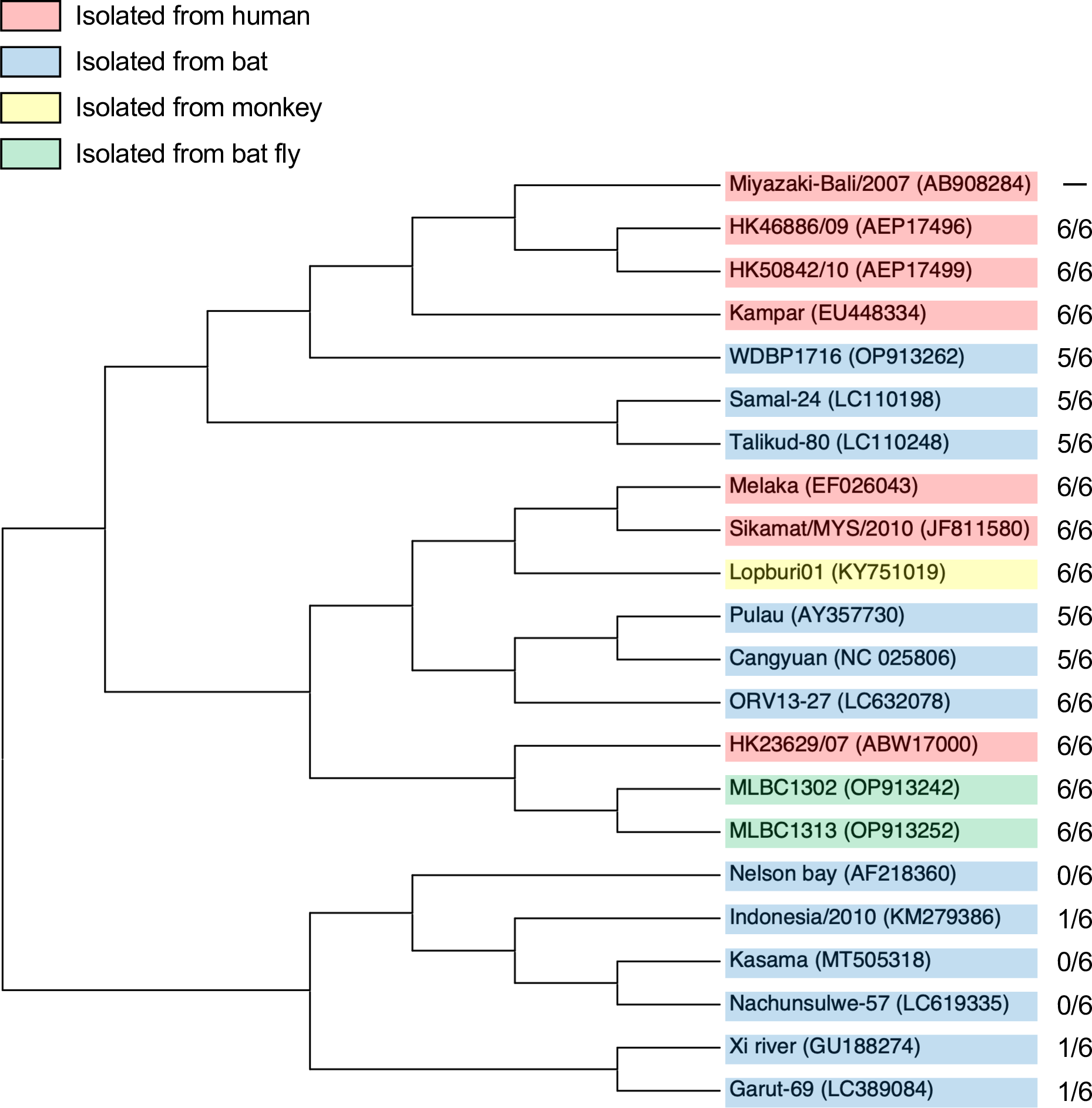

Supplement: S7 Fig — The amino acid sequences of NBV strains were used to generate the phylogenetic tree. The sequences were aligned using MUSCLE and analyzed using the maximum likelihood method with 1000 bootstrap replicates. NBV strains isolated from humans, bats, monkeys, and bat flies were highlighted in red, blue, yellow, and green, respectively. The numbers shown to the right of each strain name indicate the number of amino acids identical to those of the MB strain at the identified positions within the σC body domain. (TIF) [file ppat.1014409.s007.tif]
